# Injectable In Situ Crosslinking Hydrogel for Autologous Fat Grafting

**DOI:** 10.3390/gels9100813

**Published:** 2023-10-12

**Authors:** Kristin Oskarsdotter, Catherine T. Nordgård, Peter Apelgren, Karin Säljö, Anita A. Solbu, Edwin Eliasson, Sanna Sämfors, Henriette E. M. Sætrang, Lise Cathrine Asdahl, Eric M. Thompson, Christofer Troedsson, Stina Simonsson, Berit L. Strand, Paul Gatenholm, Lars Kölby

**Affiliations:** 1 Department of Plastic Surgery, Institute of Clinical Sciences, Sahlgrenska Academy, University of Gothenburg, 413 45 Gothenburg, Sweden; 2 Department of Biotechnology and Food Science, Norwegian Biopolymer Laboratory (NOBIPOL), Norwegian University of Science and Technology, 7491 Trondheim, Norway; 3 Department of Plastic Surgery, Region Västra Götaland, Sahlgrenska University Hospital, 413 45 Gothenburg, Sweden; 4 Department of Chemistry and Chemical Engineering, Chalmers University of Technology, 412 96 Gothenburg, Sweden; 5 DuPont Nutrition Norge AS d/b/a NovaMatrix, Postboks 223, 1377 Billingstad, Norway; 6 Ocean TuniCell AS, 5258 Blomsterdalen, Norway; 7 Department of Biological Sciences, University of Bergen, 5006 Bergen, Norway; 8 Department of Medicinal Chemistry & Cell Biology, Institution of Biomedicine, Sahlgrenska University Hospital, 405 30 Gothenburg, Sweden; 9 CELLHEAL AS, 2636 Sandvika, Norway

**Keywords:** soft tissue reconstruction, in situ crosslinking, 3D bioprinting, nanocellulose, alginate

## Abstract

Autologous fat grafting is hampered by unpredictable outcomes due to high tissue resorption. Hydrogels based on enzymatically pretreated tunicate nanocellulose (ETC) and alginate (ALG) are biocompatible, safe, and present physiochemical properties capable of promoting cell survival. Here, we compared in situ and ex situ crosslinking of ETC/ALG hydrogels combined with lipoaspirate human adipose tissue (LAT) to generate an injectable formulation capable of retaining dimensional stability in vivo. We performed in situ crosslinking using two different approaches; inducing Ca^2+^ release from CaCO_3_ microparticles (CMPs) and physiologically available Ca^2+^ in vivo. Additionally, we generated ex situ-crosslinked, 3D-bioprinted hydrogel-fat grafts. We found that in vitro optimization generated a CMP-crosslinking system with comparable stiffness to ex situ-crosslinked gels. Comparison of outcomes following in vivo injection of each respective crosslinked hydrogel revealed that after 30 days*,* in situ crosslinking generated fat grafts with less shape retention than 3D-bioprinted constructs that had undergone ex situ crosslinking. However, CMP addition improved fat-cell distribution and cell survival relative to grafts dependent on physiological Ca^2+^ alone. These findings suggested that in situ crosslinking using CMP might promote the dimensional stability of injectable fat-hydrogel grafts, although 3D bioprinting with ex situ crosslinking more effectively ensured proper shape stability in vivo.

## 1. Introduction

Autologous fat grafting is a well-established surgical technique used for restoring volumetric soft-tissue deficits and improving tissue quality, with applications in both reconstructive and cosmetic surgery [1,2,3,4,5]. The utility of this technique can be attributed to the lack of immunogenic response, relative ease of tissue isolation, and natural tissue integration as well as low donor-site morbidity [1,5]. Despite these benefits, the technique is limited due to the gradual resorption of the grafted tissue over time (range: 10–90%) [1,4,5,6,7]. This resorption makes long-term tissue retention unpredictable. Specifically, the three-dimensional (3D) shape of transplanted fat is difficult to foresee and often requires repeated procedures to obtain desired outcomes. The high tissue-resorption rate is mainly ascribed to the lack of vascularization in the grafted tissue, causing losses of cell viability and tissue volume [2,8]. There is increasing interest in strategies to counteract tissue resorption using injectable biomaterials and novel fabrication techniques [7,9,10,11,12,13,14,15]. Enzymatically pretreated tunicate nanocellulose (ETC) and alginate (ALG) are examples of biomaterials, the combination of which demonstrate utility as bioinks for extrusion-based 3D bioprinting. ETC/ALG bioinks are promising materials for clinical use in adipose tissue bioengineering due to their lack of cytotoxicity, favourable rheological properties, and gelation ability via ALG crosslinking in the presence of divalent cations (Figure S1). Moreover, the high water content of the bioink results in a high diffusion coefficient, which facilitates an efficient distribution of oxygen and nutrients throughout the material scaffold, thus increasing cell survival post-transplantation [16]. Additionally, ETC/ALG bioinks are biocompatible and do not elicit inflammatory reactions when implanted in vivo for extended periods [13]. Importantly, these hydrogels allow spontaneous vascularization when combined with lipoaspirate adipose tissue (LAT), which is a prerequisite for upscaling 3D-bioprinted fat grafts to clinical relevance [16]. These characteristics enabled pre-crosslinked 3D-bioprinted fat grafts to demonstrate excellent cell survival and shape stability for up to 150 days in vivo [17].

Application of 3D-bioprinted fat grafts is more complex as compared with conventional injection-based fat grafting. Therefore, further improvements to the fat-grafting process are necessary to ensure that 3D stability is preserved. One possible avenue would be to replace 3D bioprinting and ex situ crosslinking with direct injection of the ETC/ALG hydrogel combined with autologous LAT. Injection of these biomaterials would offer a less-invasive procedure while still possibly reaping the benefits of the hydrogel properties on cell survival and dimensional stability [9]. Such an approach infers requirements on the biomaterial with regard to both injectability and shape stability in vivo*,* with these potentially achieved through in situ crosslinking, for example through exposure to physiological concentrations of Ca^2+^. Previous studies demonstrate that gradual ALG crosslinking can be achieved using ionization of calcium carbonate (CaCO_3_) via hydrolysis of glucono-δ-lactone (gluconolactone; GDL) [18,19,20,21]. GDL hydrolyses in water, lowers the pH and increases the solubility of CaCO_3_ particles, resulting in release of calcium and bicarbonate ions that induce crosslinking and pH neutralization, respectively. This enables selective control over in situ crosslinking and could potentially simplify the grafting process relative to methods requiring pre-crosslinking.

In the present study, we investigated whether injection and in situ crosslinking of ETC/ALG hydrogels combined with LAT, using different modes of Ca^2+^ delivery, would maintain similarly high degrees of cell survival and 3D shape stability as hydrogels generated by 3D bioprinting and ex situ crosslinking.

## 2. Results

### 2.1. Rheologic Analysis of the In Situ-Crosslinked Hydrogel Systems

**Figure 1:**
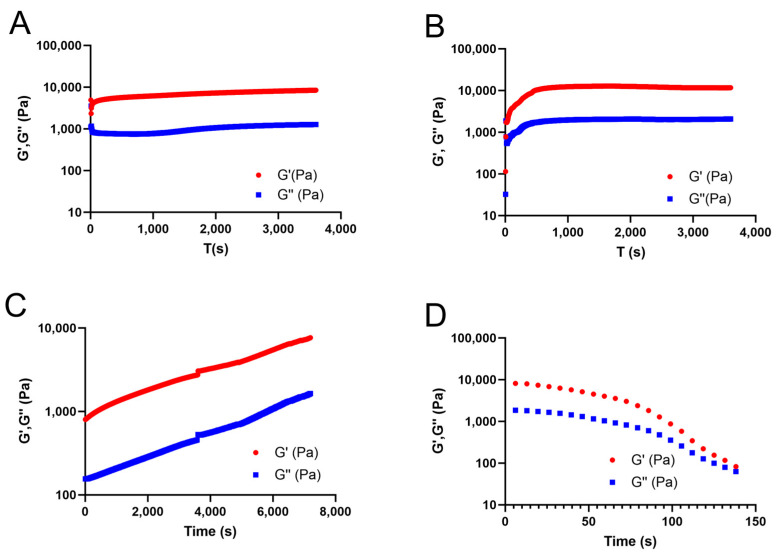
**Rheologic analysis of ETC/ALG/CMP crosslinking.** The crosslinking behavior of the ETC/ALG hydrogel through ex situ and in situ crosslinking evaluated through time-resolved shear oscillation. (**A**,**B**) The elastic modulus exhibited an increasing trend for both in situ (**A**) and ex situ (**B**) crosslinked samples. Despite an initially slower reaction, in situ crosslinking generated gels of comparable elastic moduli as those of ex situ crosslinked samples. (**C**) The same trend was observed in formulations including human LAT (LAT/ETC/ALG/CMP). (**D**) The viscoelastic properties of the in situ crosslinked samples were studied by amplitude sweep and revealed a decreasing elastic modulus according to an increasing oscillating strain. G′ (storage modulus) and G″ (loss modulus) is represented in red and blue, respectively.

Both modes of crosslinking the hydrogels without LAT performed well and generated gels with a comparable elastic modulus (Figure 1). However, initiation of in situ crosslinking (Figure 1A) was somewhat slower than that for the ex situ crosslinked samples (Figure 1B). At 1 h after initiating crosslinking, both modalities generated comparable elastic moduli, although that for the ex situ crosslinked group was slightly higher.

Oscillatory rheologic analysis using shear modulus tests to evaluate in situ crosslinking of the hydrogels including LAT (i.e., LAT/ETC/ALG/CMP), revealed an increasingly higher elastic modulus (Figure 1C,D). At 1 h after initiating crosslinking, the elastic modulus was 10-fold lower in the LAT-containing hydrogels (Figure 1C) relative to that in the pure hydrogels. However, after an additional hour of crosslinking, the elastic modulus for the LAT-containing hydrogels reached comparable levels to that in the LAT-free gels.

The viscoelastic properties of the material tested by amplitude sweep at 2 h after initiating crosslinking showed behavior typical of a physical biopolymer gel, with both the storage and loss moduli decreasing as a function of strain amplitude (Figure 1D).

### 2.2. Functional Testing of the In Situ-Crosslinked Hydrogels

**Figure 2:**
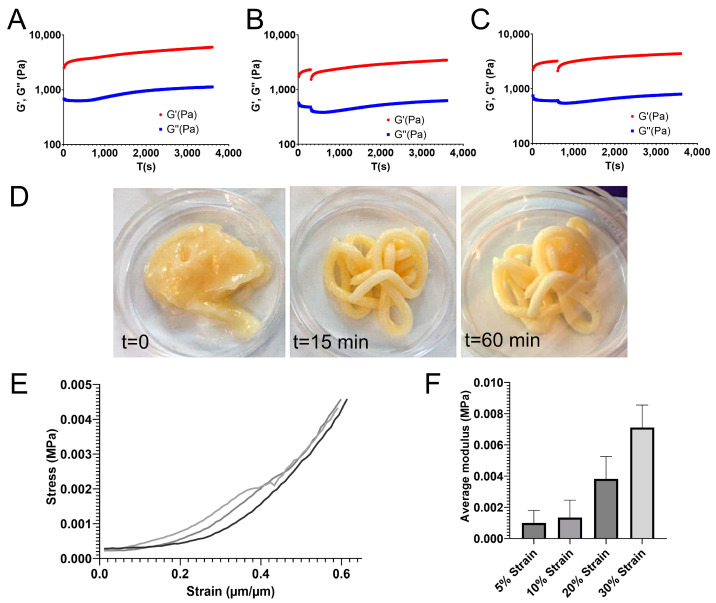
**Functional testing of the ETC/ALG/CMP hydrogel under conditions simulating injections.** (**A**–**C**) The gels were extruded at three separate time points following initiation of crosslinking by GDL addition either immediately (**A**), after 5 min (**B**), or after 10 min (**C**). The gels tolerated shear ramps for both 5- and 10-min post-GDL addition. (**D**) The extrudability of the LAT/ETC/ALG/CMP hydrogels remained for up to 1 h after GDL addition. (**E**) The stress-strain relationship (*n* = 3) of the LAT/ETC/ALG/CMP discs showed an increasing trend. (**F**) The tangent moduli calculated for selected strains ranged from 1 kPa to 7 kPa with increasing strain (5–30%). Error bars show standard deviation. G′ (storage modulus) and G″ (loss modulus) is represented in red and blue, respectively.

Injection simulations including shear ramps demonstrated that the ETC/ALG/CMP gels tolerated a shear ramp after 5 or 10 min. Additionally, the gels retained properties comparable to the undisrupted gel, suggesting that injection would be possible for up to 10 min after GDL addition (Figure 2A–C).

Functional extrusion tests that included human adipose tissue (LAT) revealed that the LAT/ETC/ALG/CMP gels remained extrudable, even in filaments, for 1 h after GDL addition (Figure 2D). Furthermore, in vitro incubation of the gel with light shaking confirmed preservation of crosslinking under physiological conditions, as the gel remained stable for up to 13 h in vitro. By contrast, LAT in the absence of ETC/ALG/CMP hydrogels was completely dispersed in the surrounding medium. 

We then tested discs comprising LAT/ETC/ALG/CMP with unconfined compression at 2 h after GDL addition. The calculated stress measured for the discs (*n* = 3) increased along with increasing strain (Figure 2E). The tangent modulus (defined as the slope of the stress-strain curve at the selected strain) demonstrated values ranging from 1 KPa to 7 KPa for strains from 5% to 30% of the indentation depth (Figure 2F).

### 2.3. In Vivo Experiments

#### 2.3.1. Macroscopic Assessment of the Explanted Hydrogel Grafts

**Figure 3:**
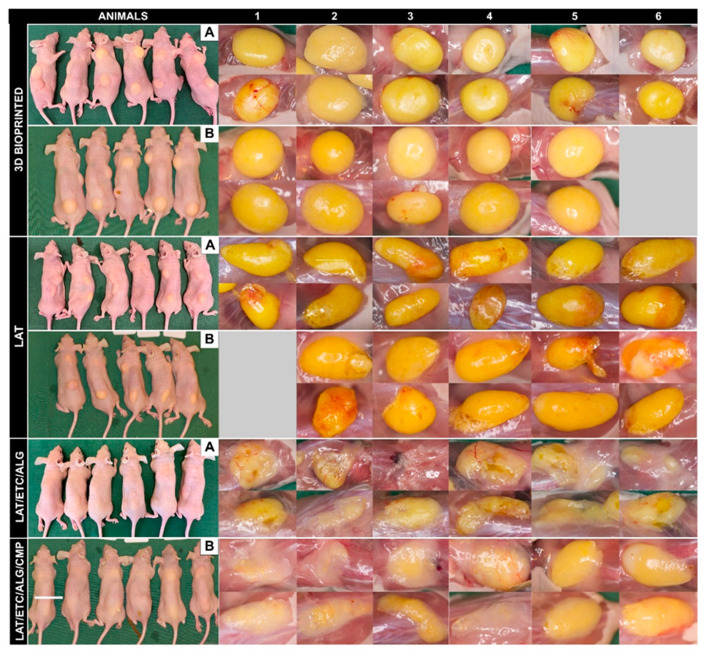
**Explantation at day 30.** (**A**,**B**) The first and second iterations of in vivo implantation, respectively. The numbered grafts (**1**–**6**) correspond to the animal from which they were explanted. Numbers in the right panels correspond to mice in the left panels (**left** to **right**). The upper and lower images denote grafts explanted from the front and back of the animal, respectively. Grey rectangles denote mice that were terminated prior to day 30.

All animals survived implantation and were alert and responsive on day 1. After 30 days, the experiment was terminated, and the grafts were exposed and assessed macroscopically (Figure 3).

The 3D-bioprinted implants retained shape and volume throughout the entire implantation period. Manual assessment confirmed the consistency as firm and stable with no signs of disintegration or other defects. All 3D-bioprinted implants were bright yellow, indicating a high content of mature adipocytes.

The injected LAT grafts were soft but stable along with a well-defined shape that showed clear borders and bright yellow color, indicating a high content of mature adipocytes. However, compared with the other groups, those with LAT injected were small in size, indicating that the injected volume was not retained over the duration of the in vivo implantation.

The injected LAT/ETC/ALG grafts (physiological Ca^2+^ crosslinking) exhibited poor retention, were soft, and did not withstand manual handling. Similarly, the LAT/ETC/ALG/CMP system produced grafts that were small and soft, although with a more well-defined shape than that of the LAT/ETC/ALG formulation. Additionally, grafts were pale, indicating a reduced content of adipocytes remaining after implantation.

In summary, the macroscopic appearance of the grafts in vivo indicated preserved fat tissue in all specimens. The best shape stability was seen in the 3D bioprinted grafts that had been crosslinked prior to implantation. Out of the two variants of in situ crosslinking, the constructs with added CMP were superior to those without CMP.

#### 2.3.2. Histologic Assessment, Adipocyte Quantification and Nanoindentation

**Figure 4:**
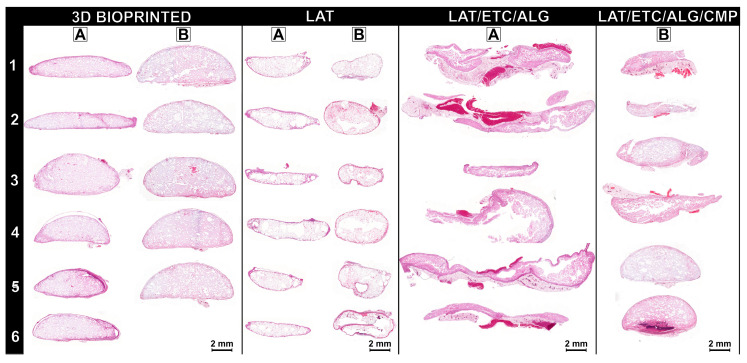
**Histologic analysis of the explanted grafts.** (**A**,**B**) Core sections from the front grafts of each animal (numbered **1**–**6**) were stained with H&E. In vivo evaluations indicated that the 3D-bioprinted grafts demonstrated well-preserved shape and contained evenly distributed adipocytes. By contrast, the injected LAT grafts were smaller and contained adipocytes from edge to edge. Injected LAT/ETC/ALG grafts were fragmented and contained very few adipocytes. Addition of CMP/GDL resulted in improved graft preservation according to both shape and adipocyte content. Scale bar: 2 mm.

**Figure 5:**
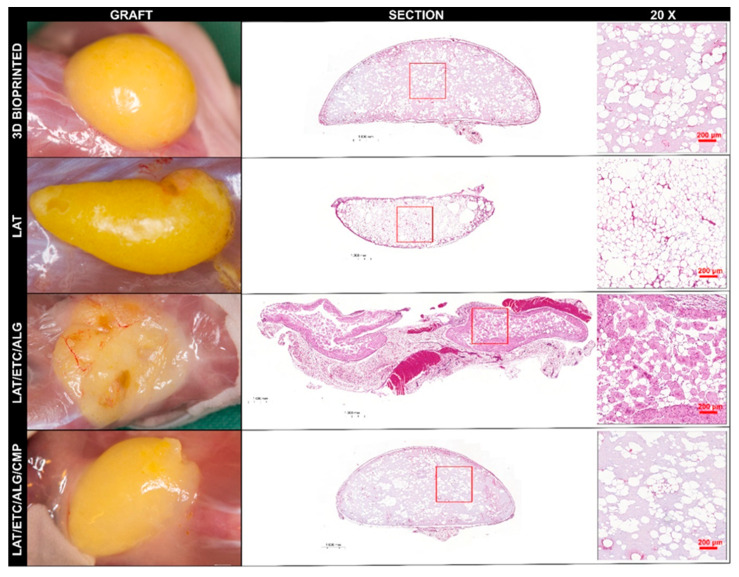
**Adipocyte distribution in the explanted grafts.** Core sections of the explanted grafts **(left**) stained with H&E and scanned to qualitatively assess adipocyte content (20× magnification; **right**) in central regions of each graft section (red squares). An even distribution of adipocytes was evident throughout the grafts in both the 3D-bioprinted (**top**), LAT (**middle**), and LAT/ETC/ALG/CMP formulations (**bottom**). The LAT/ETC/ALG grafts without CMP were less well preserved in their distribution of hydrogel and adipose cells. Scalebar: 1 mm (**middle**) and 200 µm (**right**).

The 3D-bioprinted LAT/ETC/ALG grafts exhibited high adipose tissue content after 30 days in vivo, as well as the most uniform distribution of adipose tissue and biomaterial. Comparison of individual replicates revealed that the 3D-bioprinted grafts demonstrated uniformity in both shape and morphology. The injected LAT grafts contained evenly distributed fat cells from edge to edge, and we observed no necrotic or fibrotic areas in any of the constructs. In the injected LAT/ETC/ALG grafts, adipocyte content was low, and the samples appeared broken, fragmented, and non-uniform in shape. Therefore, that formulation was not used again in the second in vivo experiment. The injected LAT/ETC/ALG/CMP grafts demonstrated improved shape, size, and distribution of adipose tissue relative to the injected LAT/ETC/ALG grafts without CMP (Figure 4). Further analysis at 20× magnification indicated an even distribution of adipocytes in the grafts that appeared to correspond well to the macroscopic appearance of the respective grafts (Figure 5).

**Figure 6:**
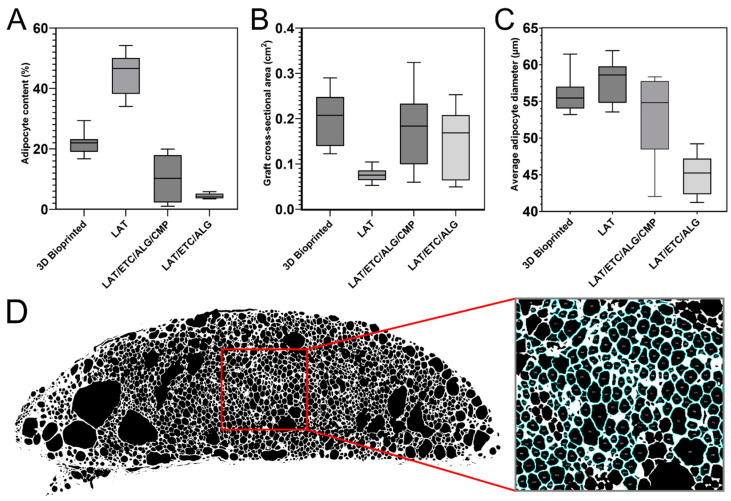
**Image analysis of the graft histology sections.** Core sections of the H&E-stained explanted grafts were converted to binary format using ImageJ software and analyzed based on adipocyte content, graft cross-sectional area, and average adipocyte diameter (2× magnification). (**A**) The 3D-bioprinted grafts showed the highest adipocyte content, with the lowest observed in the LAT/ETC/ALG grafts. (**B**) The 3D-bioprinted grafts also demonstrated the largest cross-sectional area, whereas the pure LAT grafts had the smallest. (**C**) The average adipocyte diameter was lowest for the LAT/ETC/ALG grafts and highest for the pure LAT grafts. (**D**) A representative example of a LAT histology section converted to binary format using ImageJ, with a magnified area denoted by a red square., showing counted adipocytes outlined in blue.

**Figure 7:**
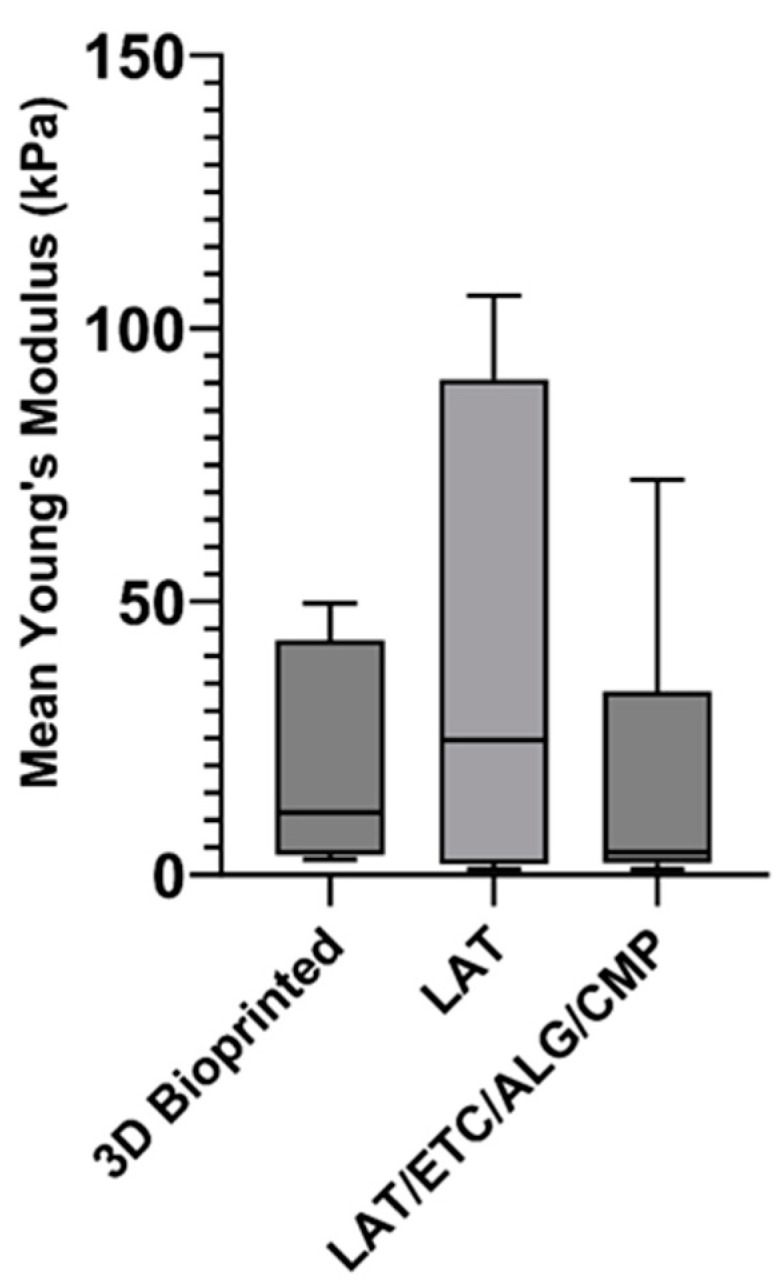
**Mean Young’s modulus for the 3D-bioprinted, CMP/GDL, and pure LAT graft explants at day 30.** Data showed significant dispersion and no significant differences between groups. Error bars show standard deviations.

Image analysis of graft sections revealed a mean adipocyte content in the 3D-bioprinted, LAT, LAT/ETC/ALG/CMP, and LAT/ETC/ALG grafts of 21.68 ± 3.51%, 44.53 ± 6.99%, 10.25 ± 8.02%, and 4.45 ± 0.89%, respectively (Figure 6A). The corresponding graft cross-sectional area was 0.199 ± 0.064 cm^2^, 0.076 ± 0.015 cm^2^, 0.178 ± 0.090 cm^2^, and 0.150 ± 0.0774 cm^2^, respectively, with a mean adipocyte diameter of 56.02 ± 2.54 µm, 57.86 ± 2.94 µm, 53.03 ± 6.07 µm, and 45.03 ± 2.82 µm, respectively (Figure 6B,C). Furthermore, nanoindentation experiments revealed no significant differences in stiffness between any of the groups (Figure 7).

In summary, the histologic assessment of the explanted grafts showed an even distribution of fat cells in the 3D bioprinted grafts, pure LAT and the LAT/ETC/ALG/CMP grafts. The grafts without CMP were fragmented. The density of the fat cells were proportional to the volumetric ratio of fat and hydrogel. The mechanical properties were comparable irrespective of composition.

## 3. Discussion

In this study, we investigated if induction of in situ crosslinking in injectable hydrogels combined with human lipoaspirate can be used to improve the volume and shape retention of autologous fat grafts. We initially evaluated the characteristics of the CMP-crosslinking system in vitro by rheological characterization of in situ and ex situ crosslinked hydrogels. For in situ crosslinking of the hydrogel, we added GDL at 30 mM/1% alginate to induce crosslinking. Theoretically, when supplied at the required concentration, the calculated concentration of GDL should be sufficient to generate release of enough Ca^2+^ to saturate the guluronate groups of the alginate despite the limited availability of Ca^2+^ relative to that generated using an external source. Rheologic analysis confirmed this, as the in situ-crosslinking generated comparable elastic moduli to those measured in the ex situ crosslinking group. Ex situ-crosslinking was achieved by addition of CaCl_2_ solution around the perimeter of the rheometer plate. This generates a relatively small surface area for ion exchange in relation to the hydrogel volume. Therefore, slower crosslinking kinetics would be expected despite the abundance of Ca^2+^. This relationship was reflected by the slower initial crosslinking of the ex situ crosslinked hydrogel (Figure 1B). Despite this difference in initial rapid crosslinking, rheologic analyses suggested that crosslinking behavior was similar for both modalities, and that the final elastic modulus was comparable. Notably, the addition of LAT slowed the crosslinking process; however, within an additional hour, the hydrogels achieved elastic moduli comparable to those in the LAT-free gels, indicating that the LAT/ETC/ALG/CMP gels were viable for in vivo evaluation.

Functional testing in vitro involving injection simulations by time-resolved low oscillatory rheology revelated that the CMP gels remained injectable for up to 10 min after GDL addition, thereby potentially prolonging the window of use in a clinical setting. The increasing stiffness over the first 2 h of crosslinking confirmed that the CMP/GDL crosslinking system combined with LAT represents a candidate for an injectable material for in vivo applications. Moreover, we observed that the in situ crosslinking system was under dynamic physiological conditions, further suggesting the LAT/ETC/ALG/CMP system as a viable candidate for in vivo applications.

Because shear moduli were determined using low oscillation, which is disruptive and cannot be used to determine mechanical properties, we performed additional evaluations of mechanical properties using unconfined compression on 3D-bioprinted discs. During these measurements, we observed increasing stress as a function of strain. This is typically observed in flexible biopolymer gel matrices, where deformation of the material under compression reduces the steric flexibility of network elements, leading to stiffening and increases in the gradient of the stress–strain curve as the matrix becomes more resistant to further deformation. Additionally, gels under compression may lose liquid, leading to a higher effective polymer concentration in the gel and a stiffer material.

Histologic assessment of grafts after 30 days in vivo revealed that the 3D-bioprinted grafts exhibited the best retention of shape and projection among all tested formulations. The distribution of fat cells was uniform throughout the constructs, and the density of fat cells was lower than that observed in pure LAT due to dilution by the hydrogel. This was confirmed by image analysis, which indicated that the adipocyte ratio was approximately 50% lower in the 3D-bioprinted grafts relative to that in the pure LAT grafts, which corresponded to the volumetric ratio of 45% fat in the 3D-bioprinted grafts before in vivo implantation (Figure 6). Additionally, the 3D-bioprinted grafts showed a larger mean surface area and the highest mean adipocyte diameter among all hydrogel-containing grafts. The high degree of cell survival in the 3D-bioprinted grafts was likely attributed to the characteristics of the bioink, including a high diffusion coefficient that allows diffusion of oxygen and nutrients and likely contributes to cell survival in the short-term [16]. In the present study, the dimensions of the 3D-bioprinted constructs exceeded traditional diffusion limits; however, the rate of cell survival suggests the importance of this feature. Furthermore, the duration of the experiment was sufficient to resemble permanent tissue transfer. For long-term cell survival, in-growth of vessels is important. A previous study reported spontaneous vessel formation and interconnection to the host circulation, and that such a feature indicates the suitability of the particular combination of LAT/ETC/ALG [16]. By contrast, injection of pure LAT resulted in considerably smaller constructs after 30 days in vivo, resulting in the lowest graft surface area among all groups (Figure 6). Notably, we found that the fat had been partially resorbed, but that the remaining construct showed an even distribution of fat cells with very high density throughout. These findings indicated that 3D bioprinting combined with ex situ crosslinking provided a dimensionally stable construct with well-preserved shape and a high degree of cell survival. These results suggest this technology as promising for further development and future clinical applications.

For the in situ crosslinked grafts, we found that relying on physiological Ca^2+^ concentrations in vivo was insufficient in ensuring dimensional stability of LAT/ETC/ALG grafts. Since the physiological concentration of Ca^2+^ ions in vivo is ~1.2 mM, this could in theory generate a slow, extended crosslinking, offering a less invasive mode of fat grafting compared to implantation of ex situ crosslinked grafts [22]. However, these grafts were neither mechanically stable nor morphologically acceptable (Figure 3 and Figure 4). We found that by inducing in situ crosslinking through release of Ca^2+^ from CMPs, this generated a notable improvement in graft morphology (Figure 3, Figure 4 and Figure 5). Although this treatment did not attain the same uniformity in appearance as the 3D-bioprinted grafts crosslinked ex situ, these grafts surpassed both injection of LAT/ETC/ALG and pure LAT. CMP contributed to in situ crosslinking and enabled tailoring of the crosslinking kinetics, making it a promising treatment for further studies.

In summary, we showed that addition of Ca^2+^-releasing agents to adipose-containing nanocellulose-alginate hydrogels increased the rate and quality of in situ crosslinking relative to in situ crosslinking dependent on physiological Ca^2+^. These improvements included enhanced shape retention and the distribution of adipose cells in the fat-hydrogel grafts. These findings suggest that in vivo injection and in situ crosslinking could become an alternative for soft tissue reconstruction.

## 4. Materials and Methods

### 4.1. Lipoaspirate Adipose Tissue Isolation and Processing

Human adipose tissue was isolated from three healthy female donors (ages 58, 46, and 43 years, respectively). Liposuction was performed in the abdominal and flank regions using a 6-mm cannula and Klein’s tumescent solution. Excess fluid was removed by decantation, and the resulting tissue was processed using the Lipogems 240 device (Lipogems International SpA, Milan, Italy) according to manufacturer instructions to generate a pure tissue fraction devoid of excess blood and debris. Briefly, adipose tissue was injected in the processing canister containing 37 °C Ringer acetate (Fresenius Kabi, Bad Homburg, Germany). The tissue was processed by repeated shaking and rinsing until the aspirate appeared light yellow, and the wash liquid was clear. Excess liquid was decanted, leaving a pure yellow lipoaspirate adipose (LAT) fraction. All procedures were performed following approval and written informed consent by the Regional Ethics Committee of Gothenburg (Dnr: 624-16). This study was conducted in accordance with national guidelines and regulations.

### 4.2. Rheologic Analysis 

Commercially available Eskal 500 CaCO_3_ microparticles (CMPs; average diameter: 4.5 µm, SD 2.054 (based on the size distribution provided by the manufacturer); KSL Staubtechnik GmbH, Lauingen, Germany) were dispersed in a 3% (*w*/*v*) solution of sterile PRONOVA SLG100 sodium alginate (ALG; DuPont NovaMatrix, Sandvika, Norway) in 4.6% mannitol to a final CMP concentration of 45 mM (15 mM/1% alginate). ALG/CMP dispersions were combined with sterile enzymatically pretreated tunicate nanocellulose (ETC; Ocean TuniCell AS, Blomsterdalen, Norway) at a 3:8 volumetric ratio (corresponding to 0.82% alginate and 1.82% ETC, *w*/*v*). Freshly prepared 0.67 M GDL solution (SigmaAldrich, Darmstadt, Germany) was added to the ETC/ALG/CMP mixture to provide a final GDL concentration of 30 mM GDL/1% alginate. A sample was transferred to the rheometer plate of a Kinexus Ultra Plus rheometer (Netzsch, Selb, Germany) fitted with 40-mm diameter serrated parallel plates immediately after GDL addition. After loading, samples were standardized by shear ramp-up test (1–100 s^−1^), and crosslinking was studied by time-resolved shear oscillation tests at 1 Hz with a target strain of 0.5% for 60 min. For comparison, ex situ crosslinking by direct addition of 100 mM CaCl_2_ to the outside of the gel on the rheometer plate was evaluated using the same hydrogel combination, excluding CMP and GDL, by time-resolved shear oscillation at 1 Hz with a target strain of 0.5% for 60 min. Storage and loss moduli were calculated and graphed as a function of time. Analyses were repeated for hydrogel formulations that included LAT. Human LAT, ETC, and ALG/CMP were combined at a 45:40:15 volumetric ratio (corresponding to 0.82% alginate and 1.82% ETC, *w*/*v*). GDL (0.67 M) was added to the hydrogel at the same concentration as that for the formulation without LAT (30 mM GDL/1% alginate), and samples were loaded on the plate of a TA Discovery 3 rheometer (Waters Corp., Milford, MA, USA). Time-resolved shear oscillation tests were then performed for 120 min immediately after addition of GDL. Storage and loss moduli were calculated and graphed as a function of time. At 2 h after GDL addition to LAT/ETC/ALG/CMP, amplitude sweeps were performed to characterize the viscoelastic behaviour of the hydrogels.

### 4.3. Functional Testing 

We evaluated the rheology of the in situ crosslinked hydrogels to assess the extrudability timeframe and thus their suitability for injection. Hydrogel crosslinking was evaluated for 60 min by time-resolved low-strain oscillatory rheology (as described in Section 2.2), with the inclusion of shear ramps to simulate injection (1–100 s^−1^) at various time points. To evaluate the extrudability of the LAT/ETC/ALG/CMP hydrogels, they were extruded through a 14-G needle and visually inspected. To evaluate stability, 0.25-mL samples of LAT/ETC/ALG/CMP were extruded into 12-well plates, with pure LAT and 3D-bioprinted and crosslinked LAT/ETC/ALG grafts used as controls. Hank’s buffered saline solution (HBSS) was added to each well, and the samples were incubated at 37 °C with shaking and assessed visually after 13 h.

### 4.4. Unconfined Compression of the In Situ-Crosslinked Hydrogels after GDL Addition

Discs (d = 10 mm; h = 1 mm) of LAT/ETC/ALG/CMP containing GDL were 3D printed using a BIOX 3D bioprinter (Cellink, Gothenburg, Sweden) at room temperature with an 18-G conical nozzle at 9 kPa to 11 kPa with a printing speed of 10 mm/s. The discs were tested by unconfined compression using a TA Discovery 3 rheometer (Waters Corp.) 2 h after printing to analyze the mechanical properties of the crosslinked material. Discs were subjected to a constant displacement rate. To convert force versus displacement to stress and strain, dimensional measurements of the samples were collected prior to performing the compression tests. To calculate stress, the force was divided by the original cross-sectional area. To calculate strain, the displacement values were divided by the original disc height and expressed as a percentage. The tangent modulus (defined as the slope of the stress-strain curve at the selected strain) was calculated.

### 4.5. In Vivo Evaluation

#### 4.5.1. Preparation of Hydrogel Grafts

LAT/ETC/ALG (45:40:15, *v*/*v*) was prepared for three different applications: injection with and without CMP+GDL and as a bioink for 3D bioprinting. Half-spheres (∅ = 10 mm, h = 3 mm) were designed using Fusion 360 CAD modeling software (version 2.0.17453; Autodesk, San Rafael, CA, USA). Half-spheres were 3D bioprinted at room temperature using the same parameter settings as described in Section 2.3. Printed half-spheres were crosslinked for 10 min at room temperature in 100 mM sterile CaCl_2_ solution (Merck, Darmstadt, Germany), followed by rinsing in HBSS supplemented with 1 mM CaCl_2_, in which it was also stored prior to implantation. A schematic presentation of the variants of crosslinking evaluated in vivo is presented in Figure S2.

#### 4.5.2. Experimental Design

We performed two consecutive animal experiments to assess the different approaches in vivo. In the first experiment, implantation of 3D-bioprinted grafts were compared with injection of LAT and injection of LAT/ETC/ALG (45:40:15, *v*/*v*) without ex situ crosslinking. In the second experiment, 3D-bioprinted grafts were compared with injection of LAT and injection of LAT/ETC/ALG/CMP.

#### 4.5.3. Animals

In vivo evaluations were performed using female Balb/C mice (aged 8–10 weeks; Scanbur, Karlslunde, Denmark) following approval by the Ethics Committee for Animal Experiments at Sahlgrenska University Hospital (University of Gothenburg, Gothenburg, Sweden: Dnr 119-2015). All animal experiments were performed in accordance with institutional, national, and European guidelines and regulations at the core facility for experimental biomedicine at the University of Gothenburg.

Animals were anesthetized in a gas chamber using isoflurane (4% induction, 2% maintenance; air flow: 2 L/min), and two dorsal incisions were made with a scalpel. The incision length was kept to a minimum: 2 mm for the injectable inks and 6 mm to 7 mm for the 3D-bioprinted constructs. Through each incision, 250-µL hydrogel was injected subcutaneously using a 14-G cannula, producing a final implanted hydrogel volume of 2 × 250 µL/animal (*n* = 5–6). For the 3D-bioprinted half-spheres, one construct was inserted in each incision. After injection or implantation, the incisions were closed using polyglactin (5–0 Vicryl Rapide; Ethicon Inc., Raritan, NJ, USA) sutures and dressed with sterile wound tape. No antibiotics were used for the duration of the in vivo studies. On day 30, animals were euthanized by cervical dislocation, and the grafts were exposed through surgical dissection. The explanted grafts from the neck region were immediately fixed in 4% buffered formaldehyde solution supplemented with 20 mM CaCl2 at 4 °C. Grafts from the lumbar region were subjected to mechanical assessment by nanoindentation directly after explantation.

#### 4.5.4. Evaluation of the Explanted Grafts

Briefly, explanted grafts were fixed in 4% buffered formaldehyde and dehydrated, paraffin embedded, and sectioned (5 µm). Deparaffinized sections were stained with hematoxylin and eosin (H&E), and sections from the core of each graft were mounted and scanned using a CIS VCC-FC60FR19CL camera and Plan Apochromat 20× objective (Zeiss, Oberkochen, Germany). Adipocytes were counted and characterized by image analysis of the scanned central sections using ImageJ software (version 1.53t; National Institutes of Health, Bethesda, MD, USA) using the “Analyze Particles” function [23,24]. Briefly, the imported scans were converted to binary format, in which empty areas (adipocyte lipid droplets) were black, and surrounding material was white. The black particles were then counted and measured according to the following inclusion criteria: (1) diameter >20 µm and <140 µm, and (2) circularity between 0.35 and 1.0. The total fat content of each graft was then calculated as the ratio between total adipocyte surface area and the graft cross-sectional area and expressed as a percentage. All calculations were performed using Excel software (v.2108; Microsoft Corp., Redmond, WA, USA). Graft stiffness after 30 days in vivo was measured by nanoindentation (Piuma; Optics11 Life, Amsterdam, The Netherlands). Indentations were performed at room temperature in air using a probe (tip radius: 26 nm; stiffness: 3.67 N/m), with measurements taken at nine different points for each graft. All measurements were performed in indentation mode with an indentation depth of 1 mm. The data were fitted according to a Hertz model, and the effective Young’s modulus (E_eff_) of each graft was calculated.

## Data Availability

The data that support the findings of this study are available upon reasonable request from the authors.

## Supplementary Materials

The following supporting information can be downloaded at: https://www.mdpi.com/article/10.3390/gels9100813/s1.
