# Supplementary material for: Injectable In Situ Crosslinking Hydrogel for Autologous Fat Grafting
Source: Gels. 2023 Oct 12;9(10):813. doi: 10.3390/gels9100813 (PMC10606883; doi:10.3390/gels9100813)
Supplement: Supplementary file 1 [file gels-09-00813-s001.zip › gels-2639303-supplementary.pdf]

Supplementary information

Injectable *in situ* crosslinking hydrogel for autologous fat grafting

Kristin Oskarsdotter[1], Catherine T. Nordgård[2], Peter Apelgren[1,3], Karin Säljö[1,3], Anita A. Solbu[2], Edwin Eliasson[4], Sanna Sämfors[4], Henriette E. M. Sætrang[5], Lise Cathrine Asdahl[5], Eric M. Thompson[6,7], Christofer Troedsson[6], Stina Simonsson[8], Berit L. Strand[2], Paul Gatenholm[9], Lars Kölby[1,3]*

[1] Department of Plastic Surgery, Institute of Clinical Sciences, Sahlgrenska Academy, University of Gothenburg, Gothenburg, Sweden

[2] NOBIPOL, Department of Biotechnology and Food Science, NTNU, Norway

[3] Region Västra Götaland, Sahlgrenska University Hospital, Department of Plastic Surgery, Gothenburg, Sweden

[4] Department of Chemistry and Chemical Engineering, Chalmers University of Technology, Gothenburg, Sweden

[5] DuPont Nutrition Norge AS d/b/a NovaMatrix, Norway (IFF H&N Norway from November 2023)

[6] Ocean TuniCell AS, N-5258 Blomsterdalen, Norway

[7] Department of Biological Sciences, University of Bergen, N-5006 Bergen, Norway

[8] Department of Medicinal Chemistry & Cell Biology, Institution of Biomedicine, Sahlgrenska University Hospital, Gothenburg

[9] CELLHEAL AS, Sandvika, Norway

*** Corresponding author**

Lars Kölby MD, PhD

Professor of Plastic Surgery

University of Gothenburg, Institute for Clinical Sciences, Department of Plastic Surgery

Gröna stråket 8, Sahlgrenska University Hospital, SE-413 45 Gothenburg, Sweden

Tel.: +46-70-6451155

E-mail: lars.kolby@surgery.gu.se

Abbreviations: 3D; three dimensional; ALG; alginate solution; CMP; calcium carbonate microparticles; ETC; enzymatically pre-treated tunicate nanocellulose; LAT; lipoaspirate adipose tissue

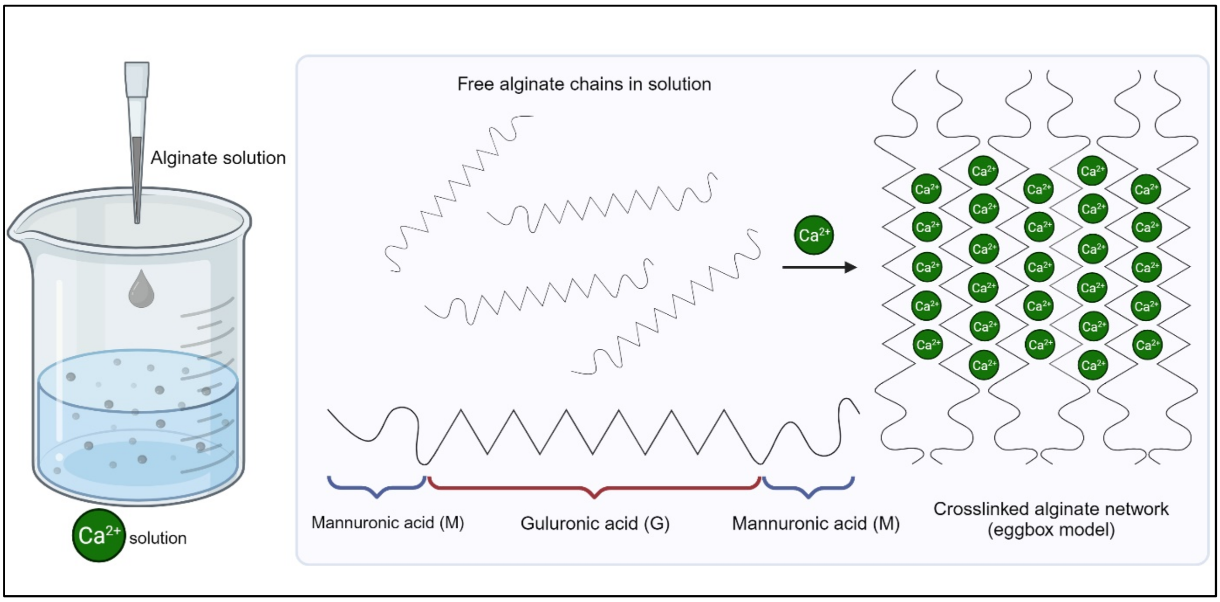

Figure S1. Schematic illustration of the molecular structure of alginate and gelation mechanism. The polysaccharide alginate are linear block copolymers consisting of linked mannuronic acid- and guluronic acid residues, denoted M and G-blocks. Alginate crosslinking can be achieved through addition of divalent cations such as Ca^{2+}. The crosslinking mechanism is believed to occur due to Ca^{2+} binding to the G-blocks of the alginate chain. Adjacent G-blocks of separate chains then form intermolecular junctions according to the eggbox model, creating a gel network. Figure created with BioRender.com.

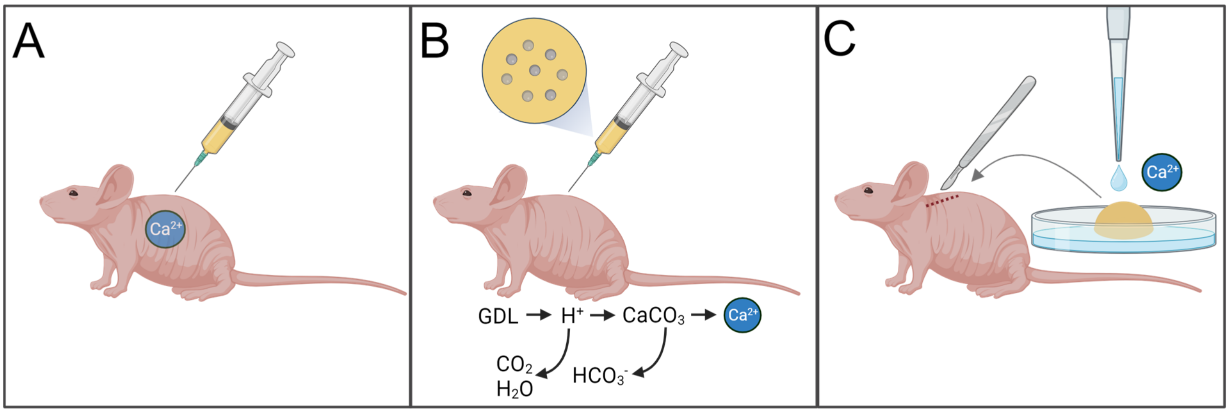

Figure S2. Schematic illustration of the delivery and crosslinking mechanisms of LAT/ETC/ALG. Three different Ca^{2+} delivery strategies were studied in a nude mouse model; injection and in situ crosslinking by physiologically available Ca^{2+} (A), injection of CMP-laden hydrogel and mechanism of Ca^{2+} release from CMP by GDL addition (B), and implantation of 3D bioprinted hydrogel grafts with Ca^{2+} supplied from an external source prior to implantation (C). Figure created with BioRender.com.
